# Supplementary material for: Facile Synthesis of NixCo3−xS4 Microspheres for High-Performance Supercapacitors and Alkaline Aqueous Rechargeable NiCo-Zn Batteries
Source: Nanomaterials (Basel). 2022 Aug 30;12(17):2994. doi: 10.3390/nano12172994 (PMC9457657; doi:10.3390/nano12172994)
Supplement: Supplementary file 1 [file nanomaterials-12-02994-s001.zip › nanomaterials-1857881-supplementary.pdf]

Supplementary Materials

Facile Synthesis of $Ni_xCo_{3-x}S_4$ Microspheres for High-Performance Supercapacitors and Alkaline Aqueous Rechargeable NiCo-Zn Batteries

Daojun Zhang [1,*], Bei Jiang [1], Chengxiang Li [1], Hao Bian [1], Yang Liu [1], Yingping Bu [1,2], Renchun Zhang [1] and Jingchao Zhang [1,*]

[1] Henan Key Laboratory of New Optoelectronic Functional Materials, College of Chemistry and Chemical Engineering, Anyang Normal University, Anyang 455000, China
[2] College of Chemistry, Zhengzhou University, Zhengzhou 450001, China
* Correspondence: zhangdj0410@126.com (D.Z.); zjc19830618@126.com (J.Z.); Tel.: +86-372-2900-040 (D.Z.)

Calculations

The specific capacitance (C_s) of $Ni_xCo_{3-x}S_4$ (x=0.9, 1.8, 2.5) electrodes using as supercapacitors can be calculated by

$$C_S\ (\mathrm{F \cdot g^{-1}}) = \frac{I\Delta t}{m\Delta V} \qquad \text{(S1)}$$

Where, *m* represents the mass of active material (g), I,Δt,ΔV is current (A), discharge time (s), and discharge potential window (V), respectively.

The specific capacity (C_m), energy density (E) and power density (P) of $Ni_{2.5}Co_{0.5}S_4$-350//Zn battery were calculated as follows:

$$C_m\ (\mathrm{mAh \cdot g^{-1}}) = \frac{I\Delta t}{m} \qquad \text{(S2)}$$

Where m, I, andΔt is the mass of active material in cathode (g), applied discharge current (A), and discharge time (h), respectively.

$$E\ (\mathrm{Wh \cdot kg^{-1}}) = C_m V \qquad \text{(S3)}$$

$$P\ (\mathrm{kW \cdot kg^{-1}}) = \frac{E}{1000 \times \Delta t} \qquad \text{(S4)}$$

Where C_m (mAh g^{-1}), V (V) represent the specific capacity of $Ni_{2.5}Co_{0.5}S_4$-350//Zn battery and the corresponding discharge plateau.

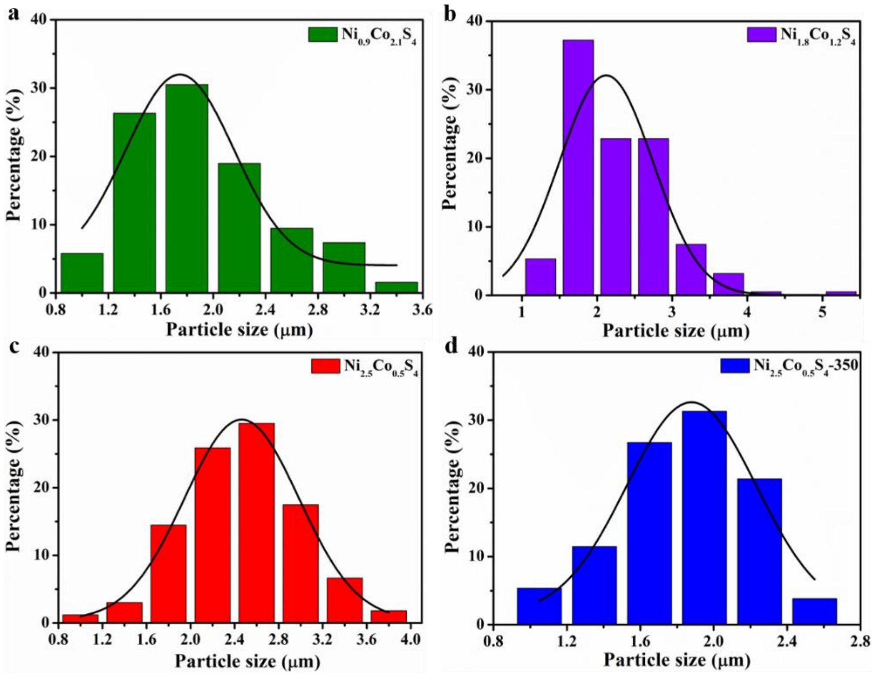

Figure S1. Size distribution for $Ni_xCo_{3-x}S_4$ series samples (a) $Ni_{0.9}Co_{2.1}S_4$, (b) $Ni_{1.8}Co_{1.2}S_4$, (c) $Ni_{2.5}Co_{0.5}S_4$, and (d) $Ni_{2.5}Co_{0.5}S_4$-350.

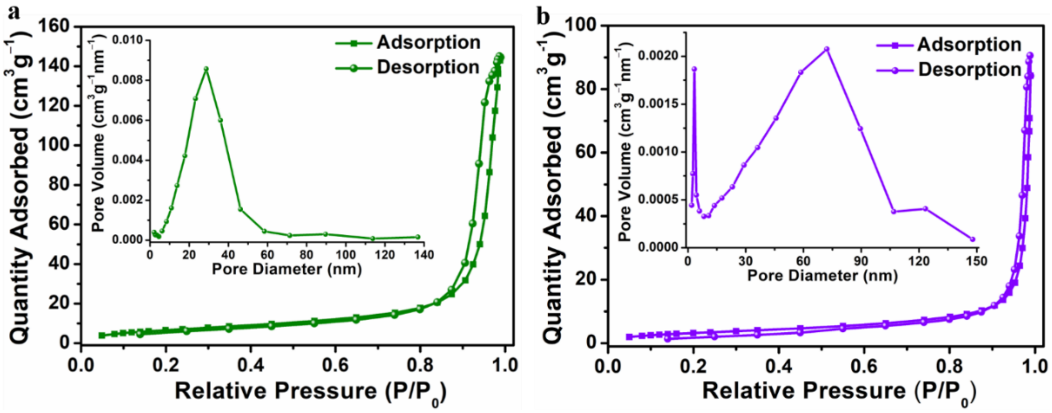

Figure S2. Isothermal plot and pore size distribution of (a) $Ni_{0.9}Co_{2.1}S_4$ (25.91 m^3 g^{-1}) and (b) $Ni_{1.8}Co_{1.2}S_4$ microspheres (12.28 m^3 g^{-1}).

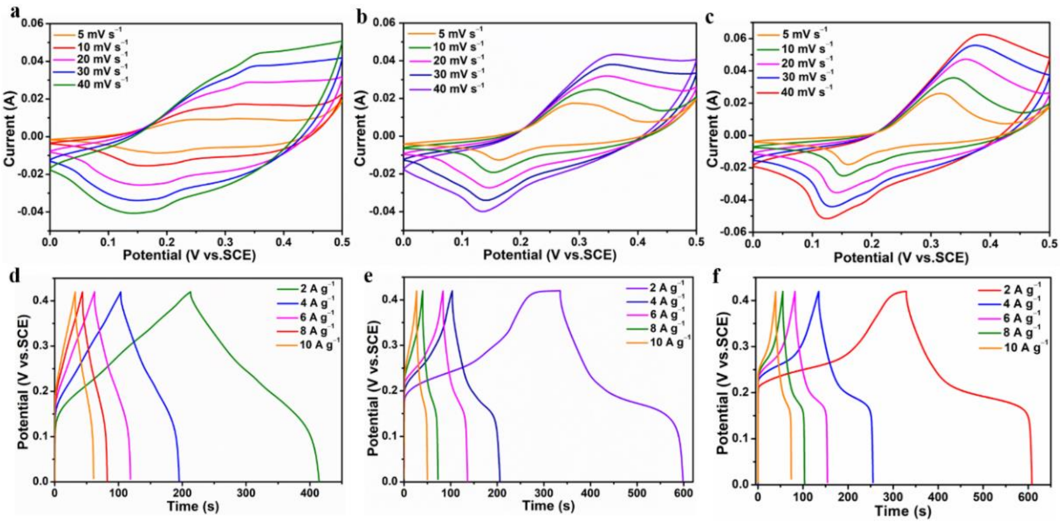

Figure S3. CV and GCD curves for $Ni_xCo_{3-x}S_4$ samples (a,d) $Ni_{0.9}Co_{2.1}S_4$, (b,e) $Ni_{1.8}Co_{1.2}S_4$, and (c,f) $Ni_{2.5}Co_{0.5}S_4$.

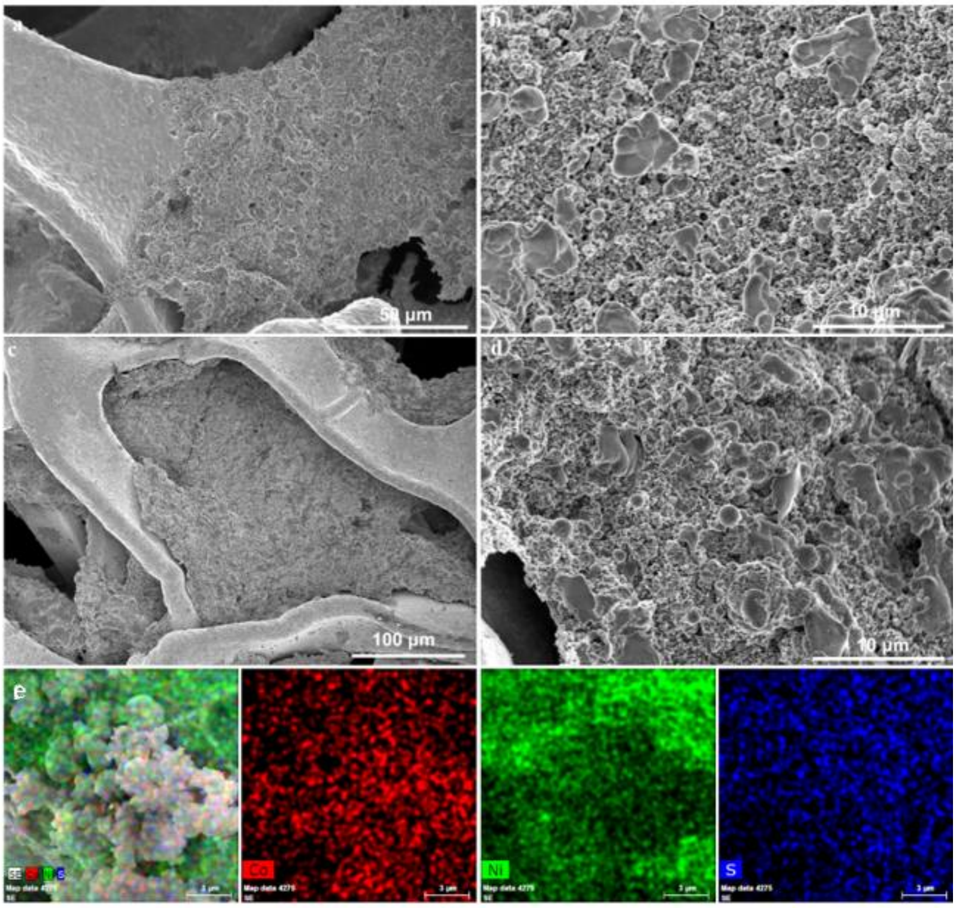

FigureS4. The SEM images of $Ni_{2.5}Co_{0.5}S_4$-350 coated on the Ni foam used as supercapacitor electrode. (a,b) before cycling tests, (c,d) after cycling tests (4 A g^{-1}, 1500 cycles), (e) the corresponding EDS mapping images after cycling tests.

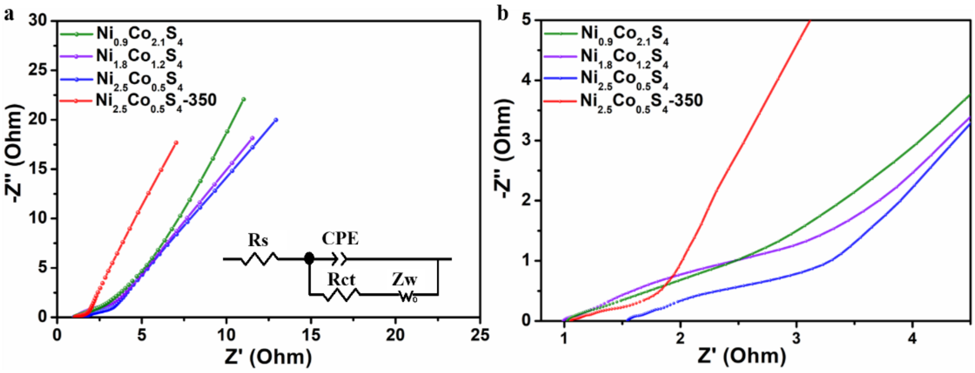

Figure S5. The Nyquist plots of $Ni_{0.9}Co_{2.1}S_4$, $Ni_{1.8}Co_{1.2}S_4$, $Ni_{2.5}Co_{0.5}S_4$ and $Ni_{2.5}Co_{0.5}S_4$-350 electrodes.

Table S1. Element composition of $Ni_xCo_{3-x}S_4$ series samples from EDS analysis.

Samples	Ni (at.%)	Co (at.%)	S (at.%)	Ni:Co:S (at.%)
$Ni_{0.9}Co_{2.1}S_4$	15.04	35.80	49.16	1:2.38:3.27
$Ni_{1.8}Co_{1.2}S_4$	31.46	19.15	49.39	1.64:1:2.58
$Ni_{2.5}Co_{0.5}S_4$	39.58	7.01	53.41	5.65:1:7.62

Table S2. The fitting resistance values of $Ni_{0.9}Co_{2.1}S_4$, $Ni_{1.8}Co_{1.2}S_4$, $Ni_{2.5}Co_{0.5}S_4$ and $Ni_{2.5}Co_{0.5}S_4$-350 electrodes.

Sample	R_s (Ohm)	R_{ct} (Ohm)	Z_w (Ohm)
$Ni_{0.9}Co_{2.1}S_4$	0.99	0.60	10.17
$Ni_{1.8}Co_{1.2}S_4$	0.98	1.20	8.80
$Ni_{2.5}Co_{0.5}S_4$	1.52	1.49	4.68
$Ni_{2.5}Co_{0.5}S_4$-350	1.03	0.40	2.03

Table S3. Comparation of $Ni_xCo_{3-x}S_4$ series samples as supercapacitors with other Co/Ni sulfide-based electrodes reported in literature.

Electrode materials	Electrolyte	Specific capacitance (F/g)	Rate performance (%)	Retention (%)	Ref
hollow C/$NiCo_2S_4$ nanosphere	6 M KOH	1545 (2 A/g)	46.6% (2-20 A/g)	90.1% (10 A/g, 6000 cycles)	34
C-$NiCo_2S_4$ hollow-nanoflake	6 M KOH	1722 (1 A/g)	39.5% (1-20 A/g)	98.8% (25 A/g, 10000 cycles)	41
onion-like $NiCo_2S_4$	6 M KOH	1016 (2 A/g)	79% (2-20 A/g)	87% (1 A/g, 10000 cycles)	42
$NiCo_2S_4$ hollow spheres	1 M KOH	756 (1 A/g)	8.7% (1-10 A/g)	300 cycles-stable value (2000 cycles)	43
nitrogen-doped carbon nanofibers@$NiCo_2S_4$	6 M KOH	1078 (1 A/g)	75.4% (1-20 A/g)<br>56.4% (1-50 A/g)	94.6% (5 A/g, 5000 cycles)	44
eggplant-derived carbon@$NiCo_2S_4$	1M KOH	1394.5 (1A/g)	80.2% (1-20 A/g)	124% (10 A/g,10000 cycles)	45
amorphous $CoNi_2S_4$ nanocages	2 M KOH	1890 (4 A/g)	81.2% (4-20 A/g)	89.9% (10 A/g, 1000 cycles)<br>71.6% (10 A/g, 5000 cycles)	46
double-shelled Zn-Co-S rhombic dodecahedral cages	6 M KOH	1266 (1 A/g)	57% (1-20 A/g)	91% (10 A/g, 10000cycles)	47
Hollow $Ni_{0.9}Co_{2.1}S_4$ microspheres	2 M KOH	959 (2 A/g)	72.9% (2-10 A/g)	101% (4 A/g, 3000 cycles)	This work
Hollow $Ni_{1.8}Co_{1.2}S_4$ microspheres	2 M KOH	1259 (2 A/g)	44.1% (2-10 A/g)	58.2% (4 A/g, 3000 cycles)	This work
$Ni_{2.5}Co_{0.5}S_4$ microspheres	2 M KOH	1335 (2 A/g)	63.3% (2-10 A/g)	50.0% (4 A/g, 3000 cycles)	This work
$Ni_{2.5}Co_{0.5}S_4$-350 microspheres	2 M KOH	2001 (1 A/g)<br>1965 (2 A/g)	89.7% (1-10 A/g)	69% (4 A/g, 1500 cycles)	This work

Table S4. Comparation of $Ni_{2.5}Co_{0.5}S_4$-350//Zn battery with other aqueous Zn ion batteries.

Battery materials	Substrate used	Electrolyte	capacity (mAh/g)	Retention (%)	Energy density (Wh/kg) @ Power density (W/kg)	Ref
$NiCo_2O_4$//Zn	carbon cloth	1 M KOH + 20 mM $Zn(Ac)_2$	183.1 (1.6 A/g)	82.7% (6.4A/g, 3500 cycles)	303.8@--	49
Ni_2P/C//Zn	carbon fiber	1 M KOH + 20 mM $Zn(Ac)_2$	176 (1 A/g)	80% (4A/g, 1500 cycles)	318@1376	50
β-$Ni(OH)_2$/CNFs//Zn	--	6 M KOH + 1 M LiOH+ PAAS + ZnO	184 (5mA/cm^2)	96% (2.54A/g, 1200 cycles)	325@1230	54
Ni-Co_9S_8-0.6//Zn	--	2 M KOH+ 0.2 M $Zn(Ac)_2$	152 (1 A/g)	69% (4A/g, 3000 cycles)	256.5@1690	55
DBS-$NiCo_2O_4$//Zn	--	6 M KOH + 0.2 M $Zn(Ac)_2$	198.6 (0.5A/g)	61.5% (1500 cycles)	326.5@822	56
P-Co_3O_4//Zn	Ni foam	1 M KOH + 0.03 M $Zn(Ac)_2$	119.4 (1 A/g)	111% (60mV/s, 5000 cycles)	193.7@1600	57
$NiCo_2S_4$@$NiMoO_4$/ Ni_2P//Zn	Ni foam	1 M KOH + 20 mM $Zn(Ac)_2$	231 (1 A/g)	122% (50mV/s, 5000 cycles)	384@ 460	58
Ni/NiO-BCF//Zn		6 M KOH + 0.5 mM $Zn(Ac)_2$	296 (0.625 A/ g)	102% (6.25 A/g, 1000 cycles).	313.4 @ 660	59
NiCo-S-2/RGO//Zn	--	6 M KOH + 0.5 M $Zn(Ac)_2$	197.1 (1 A/g)	61% (7 A/g, 1000 cycles)	333.2@1700	60
$Ni_{2.5}Co_{0.5}S_4$-350//Zn	--	2 M KOH + 0.2 M $Zn(Ac)_2$	232 (1 A/g)	54% (8 A/g, 1000 cycles)	394.6@1700	This work
